# Supplementary material for: Comparison of 24-h Diet Records, 24-h Urine, and Duplicate Diets for Estimating Dietary Intakes of Potassium, Sodium, and Iodine in Children
Source: Nutrients. 2019 Dec 3;11(12):2927. doi: 10.3390/nu11122927 (PMC6950498; doi:10.3390/nu11122927)
Supplement: Supplementary file 1 [file nutrients-11-02927-s001.pdf]

Table S1. Mean intakes of sodium, potassium, and iodine by sex and method of measurement.

Boys	N	Sodium (mg)	Potassium (mg)	Iodine (µg)
24-hour urine, mean (SD)	41	2187 (483)	1441 (389)	77 (19)
24-hour record, mean (SD)	43	2246 (146)	2111 (389)	52 (6)
Duplicate diet, mean (SD)	22	1938 (406)	2132 (560)	92 (18)
**Girls**				
24-hour urine, mean (SD)	41	2050 (384)	1387 (301)	70 (14)
24-hour record, mean (SD)	41	2199 (157)	2103 (108)	51 (6)
Duplicate diet, mean (SD)	15	1809 (405)	2231 (656)	99 (17)

Table S2. Correlations between measurement methods for sodium, potassium, and iodine

	N	Sodium (mg)	Potassium (mg)	Iodine (µg)
Spearman's correlation coefficient:				
24-hour urine & 24-hour recall	82	0.33	0.32	0.12
24-hour urine & duplicate diet	36[a]	0.44	0.35	0.65
24-hour recall & duplicate diet	37[a]	0.47	0.48	0.46
Intraclass correlation coefficient: [b]				
24-hour urine & 24-hour recall	82	0.32	0.21	0.01
24-hour urine & duplicate diet	36 [a]	0.46	0.29	0.49
24-hour recall & duplicate diet	37 [a]	0.50	0.51	0.57

[a] Sodium and potassium had two missing participants for the duplicate diet; [b] Intraclass correlation coefficient calculated using a two-way mixed-effects model for consistency of agreement for individuals.
